# *Rhodococcus* Bacteremia in Cancer Patients Is Mostly Catheter Related and Associated with Biofilm Formation

**DOI:** 10.1371/journal.pone.0032945

**Published:** 2012-03-13

**Authors:** Fadi Al Akhrass, Iba Al Wohoush, Anne-Marie Chaftari, Ruth Reitzel, Ying Jiang, Mahmoud Ghannoum, Jeffrey Tarrand, Ray Hachem, Issam Raad

**Affiliations:** 1 Department of Infectious Diseases, The University of Texas M.D. Anderson Cancer Center, Houston, Texas, United States of America; 2 Department of Microbiology, The University of Texas M.D. Anderson Cancer Center, Houston, Texas, United States of America; 3 Department of Dermatology, Center for Medical Mycology, University Hospitals Case Medical Center, Case Western Reserve University, Cleveland, Ohio, United States of America; Université d'Auvergne Clermont 1, France

## Abstract

*Rhodococcus* is an emerging cause of opportunistic infection in immunocompromised patients, most commonly causing cavitary pneumonia. It has rarely been reported as a cause of isolated bacteremia. However, the relationship between bacteremia and central venous catheter is unknown. Between 2002 and 2010, the characteristics and outcomes of seventeen cancer patients with *Rhodococcus* bacteremia and indwelling central venous catheters were evaluated. *Rhodococcus* bacteremias were for the most part (94%) central line-associated bloodstream infection (CLABSI). Most of the bacteremia isolates were *Rhodococcus equi* (82%). *Rhodococcus* isolates formed heavy microbial biofilm on the surface of polyurethane catheters, which was reduced completely or partially by antimicrobial lock solution. All CLABSI patients had successful response to catheter removal and antimicrobial therapy. *Rhodococcus* species should be added to the list of biofilm forming organisms in immunocompromised hosts and most of the *Rhodococcus* bacteremias in cancer patients are central line associated.

## Introduction

*Rhodococcus* has emerged as an opportunistic pathogen in immunocompromised patients [1], [2]. The name of *Rhodococcus* derives from the characteristic salmon- pink to red colored and teardrop shaped mucoid colonies that vary in size from 2 mm to 4 mm after 4 days of growth on solid media. *Rhodococci* are generally non-motile, intracellular aerobic, gram-positive, weakly acid-fast coccobacilli [3], [4]. Although inhalation of infected aerosols and dust particles seems to be the predominant form of transmission of *Rhodococcus* infection (>80% of cases of *Rhodococcus* have pulmonary involvement), *Rhodococcus* can also be acquired by direct inoculation into the skin and mucous membranes [5], [6]. Other routes of acquisition include nosocomial spread, human colonization and person to person transmission [7]. Furthermore, epidemiological exposure to livestock or farming environment is strongly associated with *Rhodococcus* infection in immunocompromised patients [8]. *Rhodococcu*s bacteremia is relatively rare and was perceived to be invisibly associated with concomitant cavitary lung infection [6]. Isolated bacteremia and CLABSI due to *Rhodococcus* have been rarely reported in the literature and only described in immunocompromised patients [6], [9], [10], [11], [12], [13], [14], [15]. The first objective of this study was to describe the clinical characteristics and the source and outcomes of *Rhodococcus* bacteremia in cancer patients. The second objective aimed to investigate the effect of *Rhodococcus* on the biofilm formation on central venous catheters (CVC) in a laboratory model and whether *Rhodococcus* organisms embedded in biofilm could be eradicated by an effective antimicrobial lock solution.

## Results

Descriptive statistics of patient demographics were conducted on seventeen cancer patients identified during the study period (Table S1). All patients had CVC in place at least 48 hours before the clinical presentation and blood culture sample collection. *Rhodococcus* bacteremia in our cancer patient population presented mainly with CLABSI; 16 patients (94%) had CLABSI (six of the 16 CLABSI patients fulfilled all the criteria for definite CRBSI) (Table S2) and only one patient (6%) had disseminated *Rhodococcus* bacteremia, with pneumonia as the most likely source. (*Rhodococcus equi* was recovered from bronchoalveolar lavage fluid.) Only the patient with disseminated *Rhodococcus* bacteremia with lung involvement spent 11 days in the intensive care unit for septic shock, while the other 16 patients with *Rhodococcus* CLABSI presented with few clinical manifestations only, with fever being the most common clinical complaint.

The majority of patients (65%) were initiated on combination therapy, while six patients (35%) were treated with monotherapy. Of the 17 patients, 88% underwent CVC removal, after a median duration of 43 days from the day of bacteremia (range 1–287). Only 5 patients (29%) had their CVC removed within 10 days. Patients were treated with antibiotics for a median of 11days (range, 1–90), with abatement of fever in less than 72 hours. The patients had resolution of their bacteremia after a median duration of 5 days (range 1–47 days). The median duration of stay in the hospital was 10 days (range, 2–157).

All *Rhodococcus* isolates were consistently susceptible to gentamicin and tigecycline; however, they had varying susceptibility rates to other antibiotics such as vancomycin (94%), moxifloxacin (91%), trimethoprim-sulfamethoxazole (88%), rifampin (88%), linezolid (87%), tetracycline (82%), and azithromycin (33%) and demonstrated resistance to all beta lactam antibiotics tested (penicillin, ampicillin sulbactam, and oxacillin).

### Evaluation of biofilm formation and efficacy of antimicrobial lock solution

**Figure 1:**
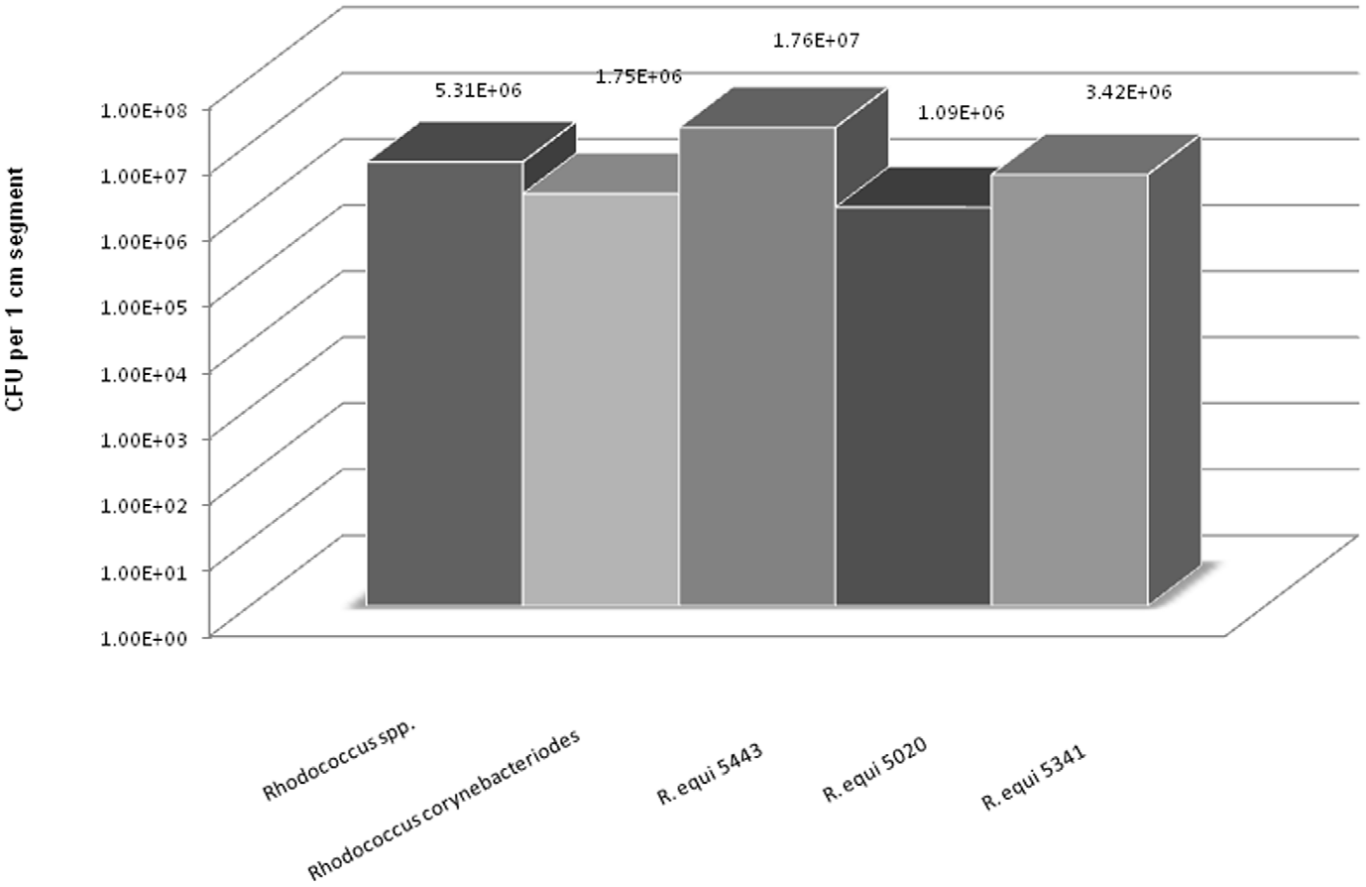
Formation of *Rhodococcus* biofilm on 1 cm polyurethane catheter segments. There was no significant difference in biofilm formation on the surface of the catheter when comparing *Rhodococcus* non-*equi* and *R. equi* isolates (p = 0.13).

All *Rhodococcus* isolates (*equi* and non-*equi*) adhered to the surface of polyurethane CVC to create extensive biofilm matrix. Quantitative culture of disrupted biofilm formation showed significant biofilm formation of approximately 10^6^ colony forming units (CFU) per 1 cm segments (range 1.09×10^6^–1.76×10^7^ CFU/segment) for *R. equi*, *Rhodococcus* spp, and *R. corynebacterioides*. (Figure 1) There was no significant difference (p = 0.13) in the amount of biofilm produced by *R. equi* (median CFU/1 cm segment: 3.85×10^6^) when compared to the non-*equi Rhodococcus* isolates (median CFU/1 cm segment: 9.63×10^5^).

**Figure 2:**
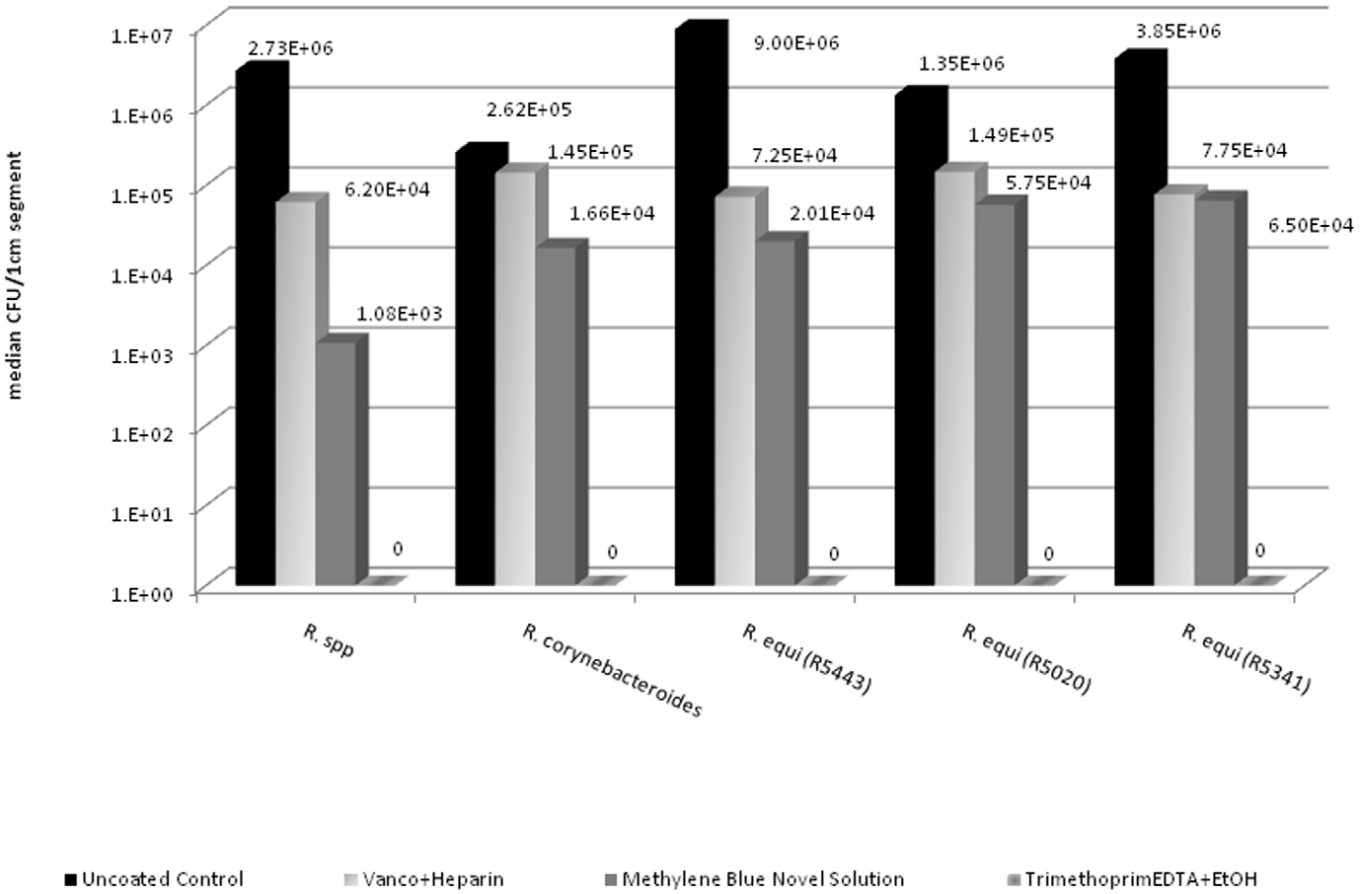
Efficacy of various lock solutions against clinical *Rhodococcus* isolates. Trimethoprim EDTA+EtOH demonstrated significant reduction in Rhodococcus biofilm formation as compared to other solutions and to control (all p-values<0.01) with a complete kill.

Of the three solutions tested trimethoprim EDTA+EtOH was significantly more effective in preventing biofilm formation when compared to other solutions and to control (all p-values<0.01) and showed complete kill when exposed to all *Rhodococcus* isolates indicating that the trimethoprim EDTA + EtOH lock solution is able to penetrate and eradicate the *Rhodococcus* in biofilm. A 1–2 log reduction was seen with the remaining lock solutions (VancoHeparin and MethBlue) indicating limited biofilm penetration and activity against *Rhodococcus* biofilm. (Figure 2)

**Figure 3:**
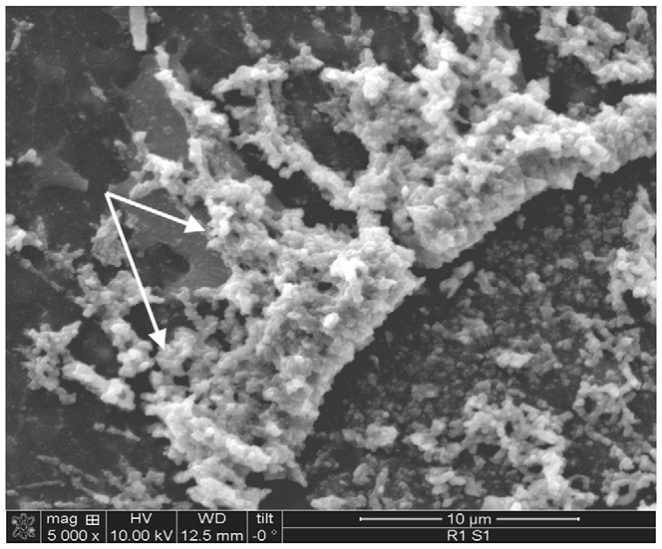
SEM of *Rhodococcus* biofilm formed on the surface of a polyurethane catheter. Image is magnified at 5000×. Extensive *Rhodococcus* gram positive rods can be seen embedded and fused together by biofilm matrix, indicated by arrows. Individual *Rhodococcus* organisms can be seen in the lower right hand corner, denoted by boxed area.

Analysis by scanning electron microscopy at 5000× magnification showed extensive biofilm formation with visible *Rhodococcus* bacterium present along with biofilm matrix. (Figure 3).

## Discussion

*Rhodococcus* CLABSI in cancer patients with advanced level of immunosuppression is not well studied and whether the CVC should be suspected as the source of a *Rhodococcus* bacteremia in the absence of apparent organ involvement has not yet been investigated.

In this study we demonstrated that *Rhodococcus* forms heavy biofilm on the surfaces of CVC polymers. Most of the *Rhodococcus* bacteremias in our cancer patients were catheter-related. Only one patient had disseminated *Rhodococcus* infection with cavitary lung disease and secondary *Rhodococcus* bacteremia. Therefore, despite their rarity, *Rhodococcus* species should be included in the differential diagnosis of organisms causing CLABSI in immunocompromised patients with advanced levels of immunosuppression.

Cancer patients depend immensely on intravascular access devices and the CVCs may become the source and focus of *Rhodococcus* bacteremia, given the ubiquitous presence of these organisms in the environment and their ability to adhere to CVCs and promote biofilm formation on the surface of the catheter. Hand contamination with *Rhodococcus* at the time of CVC manipulation may have an impact on *Rhodococcus* bacteremia. Epidemiological exposure to farming environments may clearly cause direct inoculation of the CVCs. However, in our study, information about exposures to livestock or domesticated animals such as horses and pigs was lacking. Evidence of this *Rhodococcus* biofilm formation was further shown in an in vitro biofilm model through quantitative biofilm culture and scanning electron microscope (Figures 1 and 3). Intraluminal colonization of the CVC through the formation of antibiotic-resistant biofilm matrix is the most important factor in the pathogenesis of CLABSI. The biofilm allows *Rhodococcus* species to protect themselves from the host's defenses and create a foothold from which these organisms can invade the bloodstream through the luminal or external surfaces of the CVC's intravascular segment.

Most *Rhodococcu*s human infections have been caused by *Rhodococcus equi* and only a few other species were reported to cause infections [13], [16], [17], [18]. This finding was confirmed by this current study whereby *Rhodococcus* bacteremia in cancer patients was caused mostly by *R. equi* (82%) but all species (*Rhodococcus equi* and non-*equi*) were biofilm forming (Figure 1) leading to CLABSI.

Previous studies reported that *Rhodococcus equi* infection in immunocompetent patients has a better prognosis than the disease in immunocompromised patients with a mortality of 11% vs. 20–55% respectively. [19], [20]. Recently, Yamshchikov and colleagues showed that *R. equi*-related mortality rates in organ transplant patients was 10% [4]. Unlike these previous studies, a survival rate of 100% was observed in *Rhodococcus* bacteremias in our study, thereby stressing the better prognosis associated with CLABSI in cancer patients even with advanced level of immune suppression. *Rhodococcus* CLABSI can be managed by early initiation of appropriate short course (possibly 2–3 weeks) of antibiotics active against *Rhodococcus*. In addition catheter removal might improve outcome possibly by avoiding distant recurrences and the occurrence of deep-seated infections as if the CVC is retained, the organism may persist and colonize the catheter.

In agreement with previous results, our finding also confirmed in vitro activity of aminoglycosides, glycopeptides, macrolides, fluoroquinolones, rifampicin, tetracyclines and linezolid [21]. According to these results, the antimicrobial sensitivity of *Rhodococcus* strains causing CLABSI in cancer patients did not differ from those previously reported. The most active *in vitro* antimicrobials were tigecycline and gentamicin, followed by vancomycin, fluoroquinolones, TMP-SMX, rifampin, linezolid and doxycycline. Gentamicin and glycopeptides such as vancomycin are appropriate choices.

Primary prophylaxis is not routinely recommended because no data are available to support its efficacy and because the infection is rare [6]. *Rhodococcus* bacteremia presented as a breakthrough infection while on active antimicrobial agents to which the organism was susceptible, such as fluoroquinolones, TMP-SMX and vancomycin in 45% of patients. Given the fact that *Rhodococcus* species can form an antibiotic-resistant multilayered biofilm matrix in which they embed themselves, it is not surprising that all of the breakthrough *Rhodococcus* bacteremias that occurred while on active antimicrobials were CLABSI cases. Many antibiotics are ineffective in breaking down the matrix and eradicating bacteria embedded in biofilm [22]. This is a real therapeutic and prophylactic challenge in the management of CVC in cancer patients. Hence, an effective broad spectrum antimicrobial catheter lock solution with broad spectrum activity against biofilm-forming bacteria (including *Rhodococcus*) and fungi might be the best alternative strategy for the prevention of CLABSI in cancer patients with long term indwelling CVC [23], [24] and warrants further evaluation in clinical trials.

In this current study we tested and compared the activity of various catheter lock solutions. Vancomycin/heparin and the novel methylene blue solution have been shown to reduce the risk of CLABSI caused by gram positive bacteria [25], [26]. However, trimethoprim EDTA/25%ETOH has been shown to eradicate resistant bacteria and fungi embedded in biofilm in a manner superior to the methylene blue solution as far as their activity against methicillin-resistant *Staphylococcus aureus* (MRSA), *Pseudomonas aeruginosa* and *Candida albicans* in early and mature biofilm [27], [28]. Our data support these findings whereby trimethoprim-EDTA/25% ETOH was most effective, when compared to the other solutions, in eradicating *Rhodococcus* in biofilm (Figure 2).

In conclusion, *Rhodococcus* bacteremia in cancer patients was mostly catheter related and responded well to short duration of antimicrobial therapy. Since *Rhodococcus* organisms promote extensive biofilm formation on CVC, *Rhodococcus* species must be included among the causative agents of CLABSI and the indwelling CVC should be suspected as the source of *Rhodococcus* bacteremia in cancer patients with no other apparent source for the bloodstream infection.

## Materials and Methods

Approval to conduct this study was obtained from the M. D. Anderson Cancer Center Institutional Review Board. Upon hospital admission, written consent was given by the patients for their information to be stored in the hospital database and used for research. A waiver of informed consent for this study was obtained.

### Clinical characteristics

We retrospectively identified cancer patients with *Rhodococcus* bacteremia by searching the microbiology laboratory database at The University of Texas M. D. Anderson Cancer Center, Houston, TX, (UTMDACC) from January 2002 through March 2010. Pertinent data from patients' medical records were abstracted including demographic characteristics, underlying malignancies, presence of hematopoietic stem cell transplantation, graft-versus host disease, clinical presentation, results of laboratory tests and imaging studies, concomitant infections, antibiotic therapy type and duration, and patient outcome at three month follow-up.

### Definitions

Neutropenia was defined as an absolute neutrophil count (ANC) of <500 cells/mm^3^, and lymphopenia as an absolute lymphocyte count (ALC) of <1000 cells/mm^3^. Patients with at least one set of positive blood cultures for *Rhodococcus* were defined as having *Rhodococcus* bacteremia. Once *Rhodococcus* was isolated from the blood, it was considered as a real pathogenic organism. Central line-associated bloodstream infection was diagnosed according to the Centers of Disease Control and Prevention (CDC) guidelines [29]. The diagnosis of central line associated bloodstream infection (CLABSI) was considered if the patient had a recognized pathogen cultured from one or more blood cultures (did not include organisms considered common skin contaminants) and no apparent source for the bloodstream infection except the CVC. Furthermore, CLABSI was considered as catheter-related bloodstream infection (CRBSI) if, in addition to the CDC criteria, the Infectious Disease Society of America (IDSA) definition criteria were also fulfilled, suggesting that the CVC is the culprit source of the bloodstream infection [30]. These criteria consisted of the diagnosis of CLABSI as per the CDC criteria and at least one of the following: quantitative (>10^3^ CFU/catheter segment) catheter culture, whereby the same organism was isolated from the catheter segment and peripheral blood, or by differential quantitative blood culture with simultaneous quantitative blood cultures from the CVC and the peripheral blood revealing a ratio of ≥3∶1. The diagnosis of disseminated *Rhodococcus* bacteremia required the recovery of *Rhodococcus* from a different organ noncatheter-related site (expectorated sputum, endotracheal aspiration, broncho-alveolar lavage, pleural effusion, lung tissue or skin) in the setting of clinical and radiographic evidence of organ involvement (i.e. pneumonia, skin lesions, etc.). All the patients had a CVC in place at the time of bacteremia.

### Microbiologic identification

*Rhodococcus* samples were isolated from clinical blood samples and obtained from the UTMDACC clinical microbiology laboratory. Isolates were identified on the basis of the salmon colored mucoid morphology of colonies. Speciation for the *Rhodococcus* spp and *R. corynebacteroides* was performed by the UTMDACC clinical microbiology laboratory using the 16S rRNA gene. *Rhodococcus* spp. were confirmed to be non-*equ*i and matched with several named and unnamed species such as EU741107, *R. gordoniae* W4937, *R. pyridivorans*, and *R. rhodochrous* DSM 43241. Confirmation of speciation of the *R. equi* isolates was done by PCR amplification of a 956 bp fragment of the cholesterol oxidase gene that was determined to be species specific to *R. equi* [31]. Broth micro-dilution minimal inhibitory concentration method was used for susceptibility testing of *Rhodococcus* species, according to published guidelines by the Clinical and laboratory Standards Institute (CLSI 2003). Antimicrobial response was defined as resolution or improvement of clinical manifestations, in addition to negative microbiologic findings.

### Evaluation of biofilm formation and efficacy of antimicrobial lock solution

We used a quantitative microbiological assessment and scanning electron microscope examination to assess the biofilm formation. Using a modified Kuhn's model of biofilm colonization [32], [33], *Rhodococcus* isolates, *equi* (n = 3) and non *equi* (n = 2), were tested for biofilm formation.

Each isolate was tested in sextuplet. Sterile polyurethane CVC segments were placed in 24 well tissue culture plates containing human plasma and incubated while shaking for 24 h at 37°C. The plasma was then replaced with 1 ml of 5.5×10^5^ cells/ml of *Rhodococcus* inoculum and incubated while shaking for 48 h at 37°C. Prior to exposure to catheter segments the inoculum was grown in brain heart infusion broth for 4 hrs at 37°C and diluted to 5.5×10^5^ CFU/mL. The broth was then removed, and CVC segments were washed with 1 ml of 0.9% sterile saline by shaking at 100 rpm for 30 min at 37°C. Segments were transferred into 5 ml of 0.9% sterile saline and sonicated for 15 minutes to disrupt biofilm. The resulting solution was then quantitatively cultured by making serial dilutions in 0.9% sterile saline and plating on trypticase soy agar with 5% sheep blood. All plates were incubated inverted for 48 hrs at 37°C for growth.

To evaluate the efficacy of antimicrobial lock solutions against *Rhodococcus* biofilm, all five *Rhodococcus* isolates (three *R. equi* and two non-*equi*) were exposed independently and tested in sextuplet to three antimicrobial lock solutions including, 10 mg/mL trimethoprim+30 mg/mL CaEDTA+25% EtOH (TEDTA+EtOH), 1 mg/mL vancomycin+100 uL/mL heparin (VancHeparin), and 0.05% Methylene blue+7% Sodium Citrate+0.15% methylparaben+0.015% propylparaben (MethBlue). Biofilm was formed on 1 cm segments of sterile polyurethane catheters as described above. After the washing step, segments were exposed to the three antimicrobial lock solutions for 2 hours shaking at 37°. Segments were then removed from lock solution, placed in 5 mL of 0.9% sterile saline, sonicated for 15 minutes, and quantitatively cultured as described above.

In order to visualize *Rhodococcus* biofilm, samples were evaluated in duplicate using scanning electron microscopy. One centimeter polyurethane catheter segments were exposed to *Rhodococcus* isolates as above to form biofilm. Segments were then fixed with 2% glutaraldehyde, followed by fixation with osmium tetraoxide, tannic acid, and uranyl acetate as described in previous studies [33]. Further, a series of ethanol dehydration steps were performed on the segments and then sputter coated with Au-Pd (60/40 ratio) and viewed with a model XL3C Philips microscope.

### Statistical Methods

Descriptive statistics were used to describe the basic features of the clinical data in the study. Regarding biofilm data, for each *Rhodococcus* isolate, the biofilm formation measured by CFU/1 cm segment were compared among uncoated control catheters and catheters treated with different antimicrobial lock solutions. First, a Kruskal-Wallis test was used for comparison. If a significant result (*p*<0.05) was detected, we used Wilcoxon rank sum tests for the pairwise comparisons. The α levels of the post-hoc pairwise comparisons were adjusted using a sequential Bonferroni adjustment to control type I error. Wilcoxon rank sum test was also used to compare biofilm formation between *Rhodococcus equi* and *Rhodococcus* non-*equi* isolates. All analyses were performed using SAS version 9.1 (SAS Institute, Cary, NC)

## Supporting Information

Table S1 ^a^CLABSI, central line associated blood stream infection; ^b^GVHD, graft versus host disease; ^c^HSCT, hematopoietic stem cell transplant; ^d^ICU, intensive care unit; ^e^TMP/SMX, trimethoprim-sulfamethoxazole ^*^6 patients had definite catheter-related bloodstream infection (CRBSI) ^**^Viral infection comprised Adenovirus lower respiratory tract infection in 1 (6%) and cytomegalovirus infection in 1 (6%); fungal infection comprised *Candida albicans* CLABSI in 1 (6%) and *Candida parapsilosis* CLABSI in 1 (6%); bacterial infection was represented by *Acinetobacter baumanii* bacteremia in 2 (11%) and pneumonia in 1 (6%), *Corynebacterium* CLABSI in 2 (11%), *Micrococcus* CLABSI in 1 (6%), *Pseudomonas aeruginosa* pneumonia in 1 (6%), methicillin-sensitive *Staphylococcus aureus* CLABSI in 1 (6%), Alpha-hemolytic *Streptococcus* bacteremia in 1 (6%). Patients may have coexistent viral, bacterial and fungal infections as well as multiple bacterial or fungal infections.
(DOC)

Table S2 * CLABSI, central line-associated bloodstream infection, defined according to Centers for Disease Control and Prevention; CRBSI, catheter-related bloodstream infection, defined according to Infectious Disease Society of America (10); CVC, central venous catheter; peri, peripheral; NA, not available. CVC values indicate blood for culture collected from CVC; peri values indicate blood for culture collected from peripheral vessel. All 16 patients did not have another focus of infection such as pneumonia or skin lesions (All patients had normal chest x-ray or/and CT scan of the chest at the time of enrollment).
(DOC)
