# Supplementary material for: Rhodococcus Bacteremia in Cancer Patients Is Mostly Catheter Related and Associated with Biofilm Formation
Source: PLoS One. 2012 Mar 13;7(3):e32945. doi: 10.1371/journal.pone.0032945 (PMC3302794; doi:10.1371/journal.pone.0032945)
Supplement: Table S1 — aCLABSI, central line associated blood stream infection; bGVHD, graft versus host disease; cHSCT, hematopoietic stem cell transplant; dICU, intensive care unit; eTMP/SMX, trimethoprim-sulfamethoxazole *6 patients had definite catheter-related bloodstream infection (CRBSI) **Viral infection comprised Adenovirus lower respiratory tract infection in 1 (6%) and cytomegalovirus infection in 1 (6%); fungal infection comprised Candida albicans CLABSI in 1 (6%) and Candida parapsilosis CLABSI in 1 (6%); bacterial infection was represented by Acinetobacter baumanii bacteremia in 2 (11%) and pneumonia in 1 (6%), Corynebacterium CLABSI in 2 (11%), Micrococcus CLABSI in 1 (6%), Pseudomonas aeruginosa pneumonia in 1 (6%), methicillin-sensitive Staphylococcus aureus CLABSI in 1 (6%), Alpha-hemolytic Streptococcus bacteremia in 1 (6%). Patients may have coexistent viral, bacterial and fungal infections as well as multiple bacterial or fungal infections. (DOC) [file pone.0032945.s001.doc]

**Table S1. Characteristics of patients with *Rhodococcus* bacteremia**

| Demographic and disease characteristics | Number of patients, n=17 (%) |
| --- | --- |
| Median age year (range) | 58 (7-73) |
| Male | 11 (65) |
| Hematologic malignancies | 12 (71) |
| Solid tumors | 5 (29) |
| Status of malignancy: |  |
| Active | 9 (53) |
| Remission | 8 (47) |
| Neutropenia (ANC <500 cells/μl) | 8 (47) |
| Median duration of neutropenia before diagnosis (range) | 6 (1-15) |
| Median duration of neutropenia after diagnosis (range) | 7.5 (1-30) |
| Recovery of neutropenia during treatment course | 6 (75) |
| Lymphopenia (ALC <1000 cells/μl) | 15 (88) |
| *Rhodococcus* bacteremia: |  |
| CLABSI^*^ | 16 (94) |
| Disseminated | 1 (6) |
| HSCT^c^: | 4 (24) |
| Allogeneic | 3 (75) |
| GVHD^b^ | 1 (25) |
| *Rhodococcus* species: |  |
| *Rhodococcus equi* | 14 (82) |
| *Rhodococcus* non-*equi* | 3 (18) |
| Median duration of catheter use (at time of diagnosis, range) | 99 (21-679) |
| Number of patients hospitalized | 15 (88) |
| Median duration of stay in the hospital (range) | 10 (2-157) |
| Admission to ICU^d^ after diagnosis of Rhodococcus bacteremia | 1 (6) |
| Breakthrough infections: | 6 (45) |
| While receiving fluoroquinolones | 3 (50) |
| While receiving vancomycin | 1 (17) |
| While receiving TMP-SMX^e^ | 1 (17) |
| While receiving clindamycin | 1 (17) |
| Concomitant infection:^**^ |  |
| Viral | 2 (11) |
| Fungal | 1 (6) |
| Bacterial | 5 (30) |
| Pain/tenderness and/or purulent discharge at the CVC site | 0 (0) |
| Antibiotic monotherapy: | 6 (35) |
| Fluoroquinolones | 5 (74) |
| Vancomycin | 1 (17) |
| Combination antibiotic therapy: | 11 (65) |
| Regimens containing vancomycin | 6 (55) |
| Regimens containing carbapenems | 7 (64) |
| Vancomycin + carbapenems | 4 (36) |
| Median duration of antibiotic use (range) | 11 (1-90) |
| Central line removal | 15 (88) |
| Complete response to therapy | 17 (100) |
| Death at 3 months post Rhodococcus bacteremia diagnosis | 0 (0) |
