# Supplementary material for: Rhodococcus Bacteremia in Cancer Patients Is Mostly Catheter Related and Associated with Biofilm Formation
Source: PLoS One. 2012 Mar 13;7(3):e32945. doi: 10.1371/journal.pone.0032945 (PMC3302794; doi:10.1371/journal.pone.0032945)
Supplement: Table S2 — * CLABSI, central line-associated bloodstream infection, defined according to Centers for Disease Control and Prevention; CRBSI, catheter-related bloodstream infection, defined according to Infectious Disease Society of America (10); CVC, central venous catheter; peri, peripheral; NA, not available. CVC values indicate blood for culture collected from CVC; peri values indicate blood for culture collected from peripheral vessel. All 16 patients did not have another focus of infection such as pneumonia or skin lesions (All patients had normal chest x-ray or/and CT scan of the chest at the time of enrollment). (DOC) [file pone.0032945.s002.doc]

**Table S2. Diagnostic and microbiologic profile for 16 cases of Rhodococcus CLABSI,**

**The University of Texas MD Anderson Cancer Center, January 2002-March 2010***

| **CLABSI**  **Case no.** | **Criteria for diagnosis** | **Catheter tip culture** | **CRBSI** |
| --- | --- | --- | --- |
| 1 | Differential quantitative blood culture (CVC ≥1,000 CFU/ml; peri = 1 CFU/ml) | NA | Definite |
| 2 | Differential quantitative blood culture (CVC = 200 CFU/ml; peri = 50 CFU/ml) | Quantitative catheter segment culture = 4.10^3^ CFU/tip | Definite |
| 3 | Differential quantitative blood culture (CVC = 50 CFU/ml; peri = 3 CFU/ml) | NA | Definite |
| 4 | Differential quantitative blood culture (CVC = 500 CFU/ml; peri = 1 CFU/ml) | NA | Definite |
| 5 | Differential quantitative blood culture (CVC = 1000 CFU/ml; peri =1CFU/ml) | NA | Definite |
| 6 | Differential quantitative blood culture (CVC = 200 CFU/ml; peri =1CFU/ml) | NA | Definite |
| 7 | Positive CVC blood cultures but negative peri blood culture | NA | Probable |
| 8 | Positive CVC blood cultures but negative peri blood culture | NA | Probable |
| 9 | Positive CVC blood cultures but negative peri blood culture | NA | Probable |
| 10 | Positive CVC blood cultures but negative peri blood culture | NA | Probable |
| 11 | Positive CVC blood cultures but negative peri blood culture | NA | Probable |
| 12 | Positive CVC blood cultures but negative peri blood culture | NA | Probable |
| 13 | Positive CVC blood cultures but negative peri blood culture | NA | Probable |
| 14 | Positive CVC blood cultures but negative peri blood culture | NA | Probable |
| 15 | Positive peri blood culture but negative CVC blood culture | NA | Probable |
| 16 | Positive peri blood culture but negative CVC blood culture | NA | Probable |
